# 4221 Lower Serum TWEAK Concentration is a Biomarker for Mortality in Community Acquired Pneumonia

**DOI:** 10.1017/cts.2020.411

**Published:** 2020-07-29

**Authors:** Daniela Parra, Manuela Sáenz-Valcárcel, Laura Claverias, Sandra Trefler, María Bodí, Judith Marín-Corral, Antonio García-España, Alejandro Rodriguez, Luis Felipe Reyes

**Affiliations:** 1 Universidad de la Sabana, Chía, Colombia; 2 Universidad de la Sabana, Chia, Colombia; 3 Hospital Verge de la Cinta, Tortosa, Spain; 4 Hospital Joan XXIII /URV/IISPV/CIBERES, Tarragona, Spain; 5 Hospital del Mar /IMIM, Barcelona, Spain; 6 Unidad de bilogía celular Instituto de Investigación Sanitar

## Abstract

OBJECTIVES/GOALS: To determine the relationship among serum concentration of tumor necrosis factor (TNF)-like weak inducer of apoptosis (TWEAK) and mortality in community-acquired pneumonia (CAP) patients. METHODS/STUDY POPULATION: This is a multicenter 2-year cohort study in Spain, designed to better understand the role of sTWEAK concentrations in CAP patients. CAP patients were prospectively enrolled in two University hospitals and sTWEAK was measured within the first 24 hours of ICU admission. Samples were collected and stored for laboratory analyses. To detect sTWEAK in human samples, we used a commercially available ELISA kit following manufacturer’s instructions. Demographic patients’ characteristics and ICU mortality were prospectively collected. Descriptive statistics and logistical regressions were used to assess the proposed aims. RESULTS/ANTICIPATED RESULTS: A total of forty-three patients were included in the study (10 healthy users, 10 uninfected controls and 23 CAP patients). In comparison to healthy volunteers, patients admitted to the hospital (both, infected and non-infected) had lower level of sTWEAK. During hospital admission, 7 (17%) patients died. Patients whom died during ICU stay due to CAP, had significantly lower levels of sTWEAK when comparing with patients whom survived (Median [IQR]; 509.35 [357.49, 953.92] Vs 1103.03 [716.93, 1663.16]; p = 0.015). In contrast, patients that developed shock did not have different concentrations of sTWEAK (Median [IQR]; 1008.04 [531.87, 1390.80] Vs 1062.29 [575.24, 1598.83], p = 0.84). DISCUSSION/SIGNIFICANCE OF IMPACT: Community-acquired pneumonia (CAP) is the first cause of death in underdeveloped countries. CAP is a pulmonary infection that creates a proinflammatory environment not just locally but also systemically, secondary to upregulation of molecular cascades with a wide variety of proteins being released perpetuating this inflammation and tissue damage. Several of these molecules have been described and linked to a greater risk of inhospital complications, longer length of hospital stay and mortality. TNF-like weak inducer of apoptosis (TWEAK) is a member of the TNF-alpha superfamily, involved in immune response, cell growth, angiogenesis, NF-kB activation and apoptosis induction in tumor cells. It is known that serum-TWEAK plays a role in inflammatory processes, however, its behavior is unknown in patients with CAP. Therefore, this study aims to identify whether there is a relationship between serum concentration of TWEAK and prognosis in CAP patients. To our knowledge, this is the first study to shown that concentration of sTWEAK within the first 24 hours of ICU admission is lower in patients with CAP. Moreover, patients whom died during ICU admission due to CAP, have lower sTWEAK levels. This biomarker may identify patients at higher risk of dying due to CAP and may represent severe CAP. However, further studies are needed to confirm these findings.

